# Exposure to active and passive smoking among Greek pregnant women

**DOI:** 10.1186/s12971-016-0077-8

**Published:** 2016-04-05

**Authors:** Victoria G. Vivilaki, Athina Diamanti, Maria Tzeli, Evridiki Patelarou, Debra Bick, Sophia Papadakis, Katerina Lykeridou, Paraskevi Katsaounou

**Affiliations:** Department of Midwifery, Technological Educational Institution of Athens, Athens, Greece; GAIA Maternity Hospital, Athens, Greece; King’s College London, Florence Nightingale Faculty of Nursing and Midwifery, London, UK; Division of Prevention and Rehabilitation, University of Ottawa Heart Institute & Faculty of Medicine, University of Ottawa, Ottawa, ON Canada; Department of Critical Care and Pulmonary Services, University of Athens Medical School, Evangelismos Hospital, Athens, Greece

**Keywords:** Tobacco smoking in pregnancy, Health behaviour, Environmental tobacco smoke, Smoking cessation, Pregnancy complication, Midwives

## Abstract

**Background:**

Active smoking and exposure to passive smoke are responsible for numerous adverse pregnancy outcomes for women and their infants. The aim of this study was to explore the perceptions, attitudes, patterns of personal tobacco use and exposure to environmental smoke among a sample of pregnant women in Greece.

**Method:**

A cross sectional survey was undertaken of 300 women identified from the perinatal care records of the Maternity Departments of two hospitals in Athens between February 2013 and May 2013. Data on active and passive maternal smoking status in the first, second, and third trimesters of pregnancy, fetal and neonatal tobacco related complications, exposure to environmental tobacco smoke during pregnancy, quit attempts, behaviors towards avoiding passive smoking and beliefs towards smoking cessation during pregnancy were collected using self-administered questionnaires on the 3rd postnatal day. Women also completed the Edinburgh Postnatal Depression Scale (EPDS).

**Results:**

Of 300 women recruited to the study 48 % reported tobacco use during the first trimester of pregnancy. Amongst participants who were tobacco users, 83.3 % reported making an attempt to quit but less than half (45.1 %) were successful. Among women who continued to smoke during pregnancy the majority (55.8 %) reported that they felt unable to quit, and 9.3 % reported that they considered smoking cessation was not an important health issue for them. Participants who continued to smoke during pregnancy were more likely to report fetal (χ2 = 11.41; df = 5; *p* < 0.05) and newborn complications (χ2 = 6.41; df = 2; *p* < 0.05), including preterm birth and low birth weight. Participants who reported that their partners were smokers were more likely to smoke throughout their pregnancy (χ2 = 14.62; df = 1; *p* < 0.001). High rates of second-hand smoke exposure were reported among both smoking and non-smoking women. Pregnant smokers had significantly higher levels of postnatal depressive and anxiety symptomatology, as measured using the EPDS, than non-smokers.

**Conclusion:**

Our data supports the importance of ensuring that pregnant women, their partners and close relatives are educated on the health risks of active and passive smoking and how these could have an adverse effect to their fetus and infants, as well as the pregnant women themselves.

## Background

Eliminating active and passive maternal tobacco smoking during pregnancy are among the most important interventions to reduce the risk of adverse birth outcomes [1–5]. Previous studies have reported that tobacco smoking during pregnancy is significantly associated with increased risks of intrauterine growth retardation, preterm birth, low birth weight, miscarriage, stillbirth, congenital malformation, sudden infant death syndrome, genetic-related hereditary diseases, perinatal mortality and morbidity, short stature, cognitive delays, and neurologic disorders [2–9]. Pregnant women who smoke place themselves and their infants in a high-risk situation [2]. However, more than a third of women smokers will continue to smoke during pregnancy, despite being aware of many of the imminent risks to their infants [10].

Previous studies have demonstrated that pregnant smokers usually have partners who actively smoked during pregnancy [8, 11, 12]. The health of pregnant women and their fetuses’ is inherently threatened by both the active and passive smoking of the pregnant women’s partners or families [2, 12]. However, the effect of passive maternal smoking is not explicit enough and has not been extensively studied [9, 13, 14]. The perceptions of pregnant smokers regarding the health risks of smoking and the need to abstain from passive smoking have been described as important factors influencing a smoke-free behavior [2, 8]. A partner who continues using tobacco throughout a woman’s pregnancy is a significant prognostic factor of the current smoking status of the pregnant woman [2, 11, 15].

Recent studies have generally highlighted the need to conduct further research on the types of interventions which could be employed in order to set goals for reducing smoking in pregnancy and promote smoke-free environments, as a potential benchmark of an effective primary care system [16]. Midwives and other community health professionals need to educate, and offer support to pregnant women to stop smoking and avoid postnatal relapse among woman who have quit smoking during their pregnancy [17]. Despite the fact that pregnancy provides a ‘window of opportunity’ to encourage positive behavior change, encouraging pregnant smokers to change their health behavior may be challenging [3].

The aim of this study was to explore the perceptions, attitudes and behaviors towards active and passive maternal smoking during pregnancy of smokers, non-smokers and recent quitters in Athens, Greece. Our specific objectives were to: i) assess the proportion of women who were active smokers and exposed to second-hand smoking during pregnancy; ii) compare pregnant smokers and non-smokers in regards to postnatal depressive and anxiety symptomatology, neonatal problems, partner smoking habits and other sources of passive smoking (work, social places, car etc.); and iii) explore women’s perceptions and attitudes towards smoking during the perinatal period.

## Methods

### Instruments

A self-administered questionnaire was developed to collect baseline socio-demographic data from women on their total household income, employment status, ethnicity and age at which full-time education was completed. In addition, women’s reproductive history was also recorded, including history of previous miscarriage or pregnancy termination, family planning, type of birth and antenatal/postnatal complications, attitudes toward tobacco smoking, perceived health risk, smoking history, smoking volume (before and during the index pregnancy) and exposure to passive smoking during the index pregnancy.

### Edinburgh postnatal depression scale (EPDS)

The Edinburgh Postnatal Depression Scale (EPDS) [18] is a ten item self-report scale, consisting of statements describing depressive and anxiety symptoms experienced during the last 7 days, which can be administered at any stage following birth [19]. High EPDS scores in the first week of birth could identify women with low mood or postnatal ‘blues’, a transient psychological condition, which some studies have identified as a potential indicator of postnatal depression if symptoms persist [20]. Each item is scored on a point scale ranging from 0 to 3, depending on the severity or duration of each symptom. The Greek version of the EPDS used in this study was validated and demonstrated high internal consistency (Chronbach’s alpha = 0.804 and Guttman split-half coefficient 0.742). The Greek EPDS was significantly correlated (Pearson *r* = 0.66 *p* < 0.0005) to the validated Greek version of BDI-II (Beck Depression Inventory II) [21]. A threshold score of 8/9 fitted the model sensitivity at 76.7 % and model specificity at 68.3 % [22].

### Setting, sampling and target population

Following the pilot testing of the smoking habits questionnaire using a focus-group method, the questionnaire was administered to women who gave birth during February 2013 to May 2013 inclusive who were inpatients on the postnatal wards of two public maternity hospitals in Athens. Women were asked to complete the questionnaires on their 3rd postnatal day. Women were considered eligible to take part in the study if they met the following criteria: (1) aged 16 to 45 years; (2) fluent in spoken and written Greek; (3) healthy mother and infant following the birth; and (4) able to provide informed consent.

### Participants and data collection

**Fig. 1:**
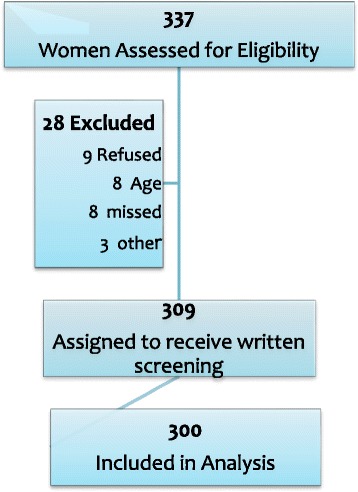
Study flow diagram

A total of 337 women were identified as eligible from the perinatal care records of the two maternity units (Fig. 1). The midwife-researchers (VV, MT) ensured there was a balance in recruitment using a calendar to recruit participants across different shifts and days of the week. More specifically, the women were recruited on a one-day a week basis in both sites (i.e. first week on a Monday, the following week on a Tuesday, the week following that on a Wednesday, etc.). This technique was employed in order to avoid bias associated with possible seasonality of smoking habits. Each recruitment day was split into three shifts (8 a.m., 4 p.m., 12 a.m.), and the first four women who had given birth after 8 a.m. were selected on one week and likewise, the first four who had given birth the following week after 4 p.m. were also selected. This ensured the reduction of possible bias related to the time of smoking.

Women were encouraged to discuss any concerns they had about their smoking status with the research midwives. In these cases women were advised that their ward and/or community midwife would be informed of their responses to the screens for smoking status, in order to refer the women to s smoking cessation support services if they wished to quit. All participants were informed verbally by the research midwives about smoking cessation health services available in the community to support them postnatally.

The research ethics boards of both hospitals approved the study (reference number #113/9-5-2012). All participants provided oral informed consent prior to enrolment. Included with the questionnaires was a cover letter explaining the purpose of the study, providing the researchers’ details and contact information, and clearly stating that all answers would be confidential and no names would ever be used in any reports presenting the study findings.

## Data analysis

Statistical analysis was performed using IBM SPSS Statistics version 20.0 and LISREL (Linear Structural Relations). The descriptive characteristics were calculated for the socio-demographic variables. The assumptions of normality, homogeneity and independent cases of the sample were checked. We used chi-square tests to investigate whether significant differences existed in maternal beliefs and attitudes toward smoking during pregnancy between groups of quitters and smokers. T-tests were carried out to compare the descriptive variables, risk perception, attitudes to smoking, smoking behaviors women who smoked during pregnancy. Reliability coefficients, as measured by Cronbach’s alpha, were calculated for the smoking questionnaire in order to assess reproducibility and consistency of the instrument; and the internal consistency of the questionnaire was also tested using Guttman split-half coefficients.

## Results

### Sample characteristics

Of the 337 women initially approached, 300 consented to take part in the study, a participation rate of 89.0 % (Fig. 1). The women had a mean age of 33.76 years (Standard Error of Mean, SEM 0.537, range 20–45 years). Socio-demographic characteristics are shown in Table 1, which indicates that 48 % of the participants were smokers at pregnancy commencement. In total, 73.7 % of the total sample reported being smoke-free during pregnancy. Among tobacco users, 83.3 % tried to quit and less than half of participants (45.1 %) were successful. Twenty-two percent of women had quit during pregnancy and 26.3 % of the participants continued to smoke during pregnancy. Among women who did not stop smoking during pregnancy, 55.8 % claimed that they could not stop smoking, another 25.6 % stated that they did not want to stop smoking and 9.3 % of women claimed that they considered smoking cessation was not an important health issue for them. The harmful effects of smoking during pregnancy on the fetus (χ2 = 11.41; df = 5; *p* < 0.05) and the newborn (χ2 = 6.41; df = 2; *p* < 0.05) were confirmed in our study. The smoking status of the partner was associated with an increased likelihood that a woman continued to smoke throughout her pregnancy (χ2 = 14.62; df = 1; *p* < 0.001).

**Table 1:** Characteristics of the 300 pregnant women sample between February 2013 to May 2013

	Smoking status during pregnancy			
	Smokers women *n* (%)	Non smokers women *n* (%)	Total *n* (%)	*P* value
Age				
16–20	9(3)	7(2.3)	16(5.3)	0.502
21–30	63(21)	60(20)	123(41)	
31–40	67(22.3)	77(25.7)	144(48)	
> 40	5(1.7)	12(4)	17(5.7)	
Nationality				
Greek	129(43)	126(42)	255(85)	0.012
Other	15(5)	30(10)	45(15)	
Education				
Elementary & junior high	6(2)	6(2)	12(4)	0.821
High School	70(23.3)	67(22.3)	137(45.7)	
University/College education	54(18)	71(23.7)	125(41.7)	
Postgraduate studies	14(4.7)	12(4)	26(8.6)	
Work status				
Housewife	64(21,3)	63(21)	127(42.3)	0.398
Public sector	20(6.7)	34(11.3)	54(18)	
Private sector	36(12)	40(13.3)	76(25.3)	
Self-employed	24(8)	19(6.3)	43(14.3)	
Gravida				
Primigravida	86(28.7)	69(23)	144(48)	0.034
Multigravida	58(19.3)	87(29)	156(52)	
Marital Status				
Married	116(38,7)	147(49)	263(87,7)	0.002
Single	23(7,7)	9(3)	32(10,7)	
Divorced	4(1,3)	0(0)	4(1,3)	
Widow	1(0,3)	0(0)	1(0.3)	
Pregnancy				
Planned	67(22.3)	99(33)	144(48)	0.003
Unplanned	77(25.7)	57(19)	156(52)	
Mode of birth				
Vaginal birth	78(26)	86(28.7)	164(54.7)	0.910
Cesarean section	66(22)	70(23.3)	136(45.3)	

### Depressive symptoms and smoking status

**Fig. 2:**
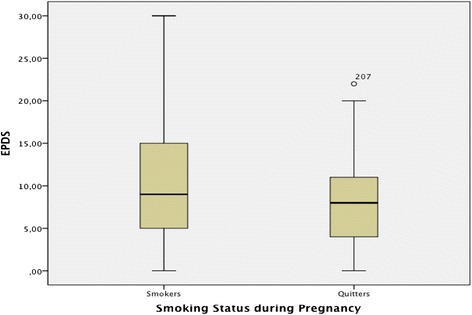
Smoking Status and Depressive symptomatology

The mean EPDS score for current tobacco users was 9.72 (SD = 6.280; Std Error Mean 0.523) and for non-smokers was 8.044 (SD = 5.178; Std Error Mean 0.414). The non-smokers group included women who had never smoked or stop smoking before pregnancy and those who quit during pregnancy. Smokers reported significantly higher levels of depressive symptomatology than non-smokers [Levene’s Test for equality of variances and homogeneity was calculated (*F* = 43.059, *P* = 0.0005) (*t* = 2.403 df = 298 Sig. (2-tailed) =0.0005] (Fig. 2).

### Maternal health behavior and attitudes regarding smoking

**Fig. 3:**
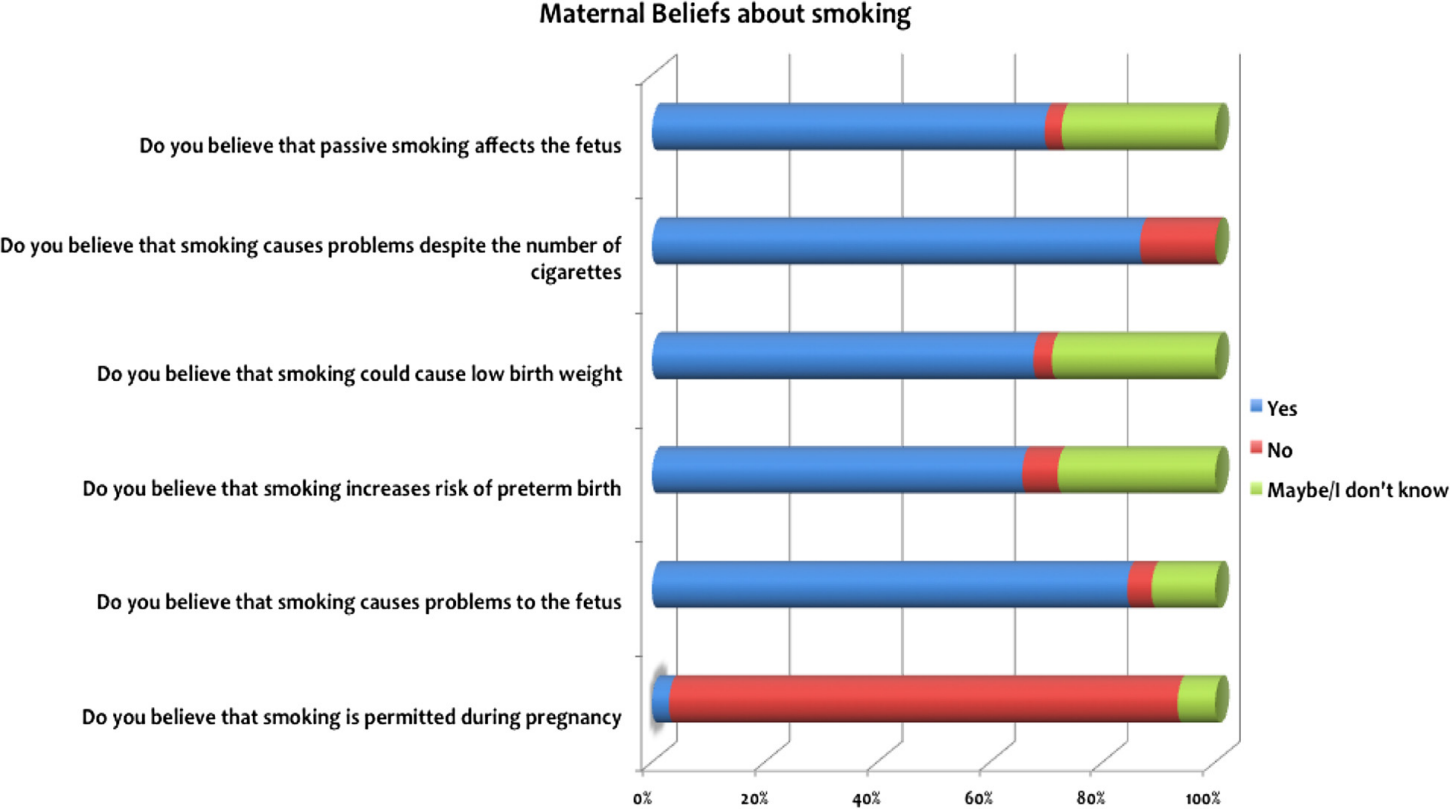
Maternal beliefs about smoking

Fetal health was a critical reason for pregnant women to quit smoking, and most of the participants who quit chose to do this when they found out they were pregnant (Fig. 3). The majority of participants who quit were aware of the threat of smoking to fetal health (*n* = 208, 84.3 %). Of the 119 (83.3 %) women who tried to reduce or quit smoking because of their pregnancy 64 women (45.1 %) succeeded and 55 (38.7 %) continued to smoke during pregnancy. A small proportion of women (2.7 %) reported that they did not understand that smoking was not recommended during pregnancy because of the high level of risk to fetal health (Table 2).

**Table 2:** Maternal perceptions and attitudes about smoking

	Smoking status during pregnancy			
	Smokers women *n* (%)	Non-smokers women *n* (%)	All women total *n* (%)	*P* value
Did you try to reduce or quit because of pregnancy?				
Yes	55 (38.7)	64 (45.1)	119 (83.3)	>0.001
No	19 (13.4)	4 (2.8)	23 (16.2)	
Do you believe that smokingis permitted during pregnancy?				
Yes	5 (1.7)	3 (1)	8 (2.7)	>0.001
No	63 (21.1)	208 (69.6)	271 (90.7)	
Do you believe that smoking causes problems to the fetus?				
Yes	44 (14.7)	208 (84.3)	252 (99)	>0.001
No	11 (3.7)	2 (9.6)	13 (13.3)	
Do you believe that smoking causes problems despite the number of cigarettes?				
Yes	54 (18.2)	203 (68.4)	257 (86.6)	>0.001
No	24 (8.1)	16 (5.4)	40 (13.5)	
Do you believe that passive smoking affects the fetus?				
Yes	38 (12.7)	170 (56.9)	208 (69.6)	>0.001
No	6 (2)	3 (1)	9 (3)	

### Passive smoking

**Fig. 4:**
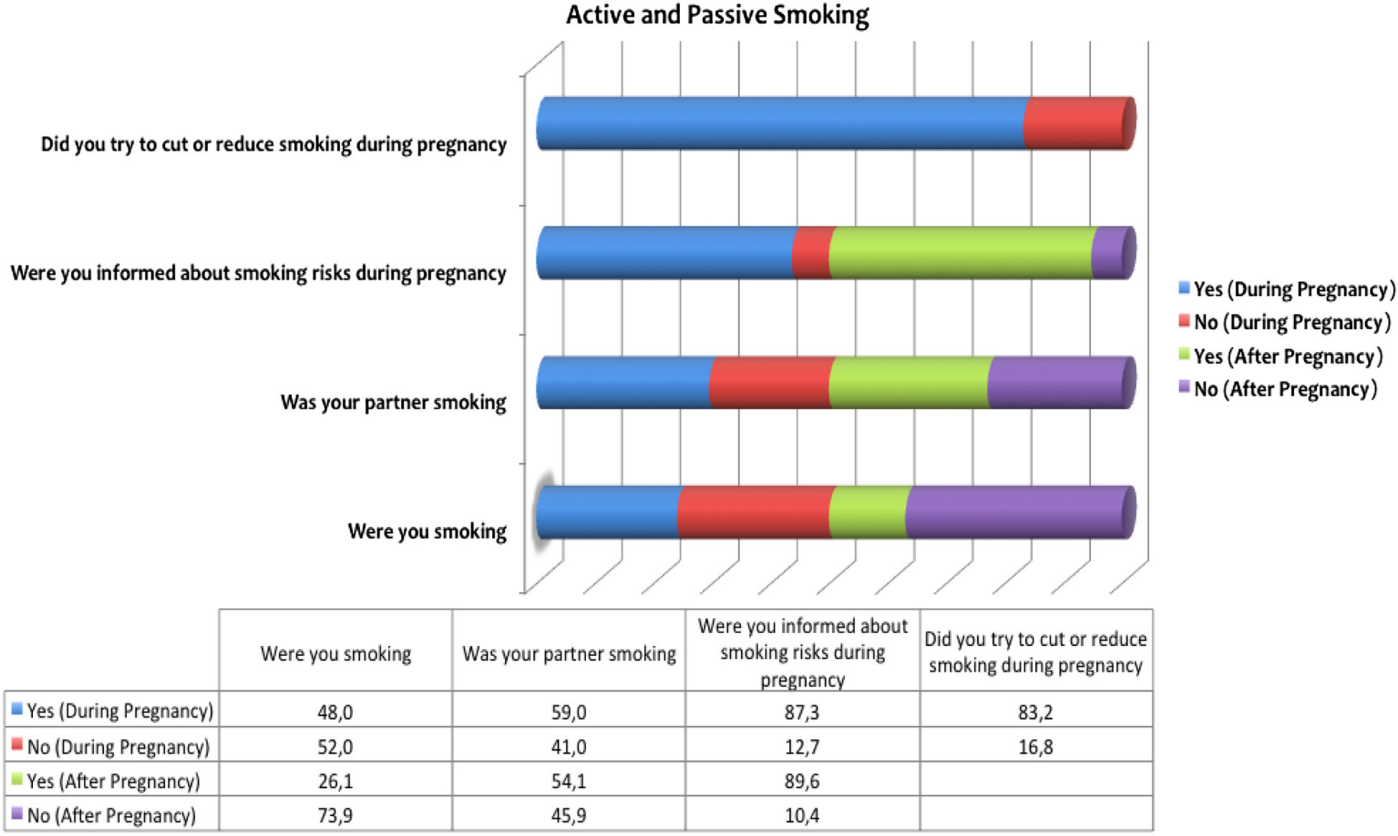
Active and passive smoking during and after pregnancy

**Fig. 5:**
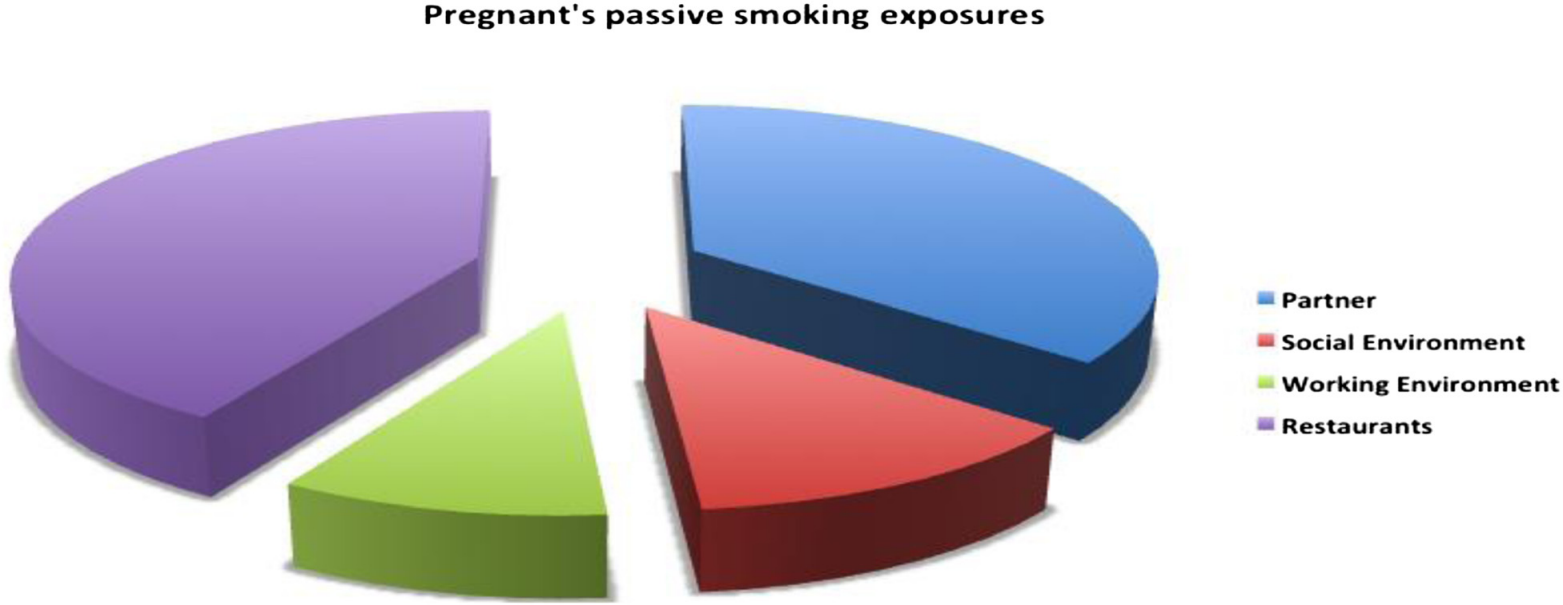
Pregnant’s passive smoking exposures

Table 3 shows the exposure of the participants to passive smoking. Forty-two percent of participants lived with a partner who smoked; 13.5 % of the participants lived with relatives who smoked; 9.7 % were exposed to smoke in their work environment; and 34.7 % reported being regularly exposed to second hand smoke in restaurants. Among the participants whose partners were smokers, 15.8 % were active smokers and 26.3 % non-smokers. (Figs. 4 and 5).

**Table 3:** Women’s passive smoking exposures during pregnancy

	Smoking status during pregnancy			
	Smokers women *n* (%)	Non-smokers women *n* (%)	Total *n* (%)	*P* value
Sources of passive smoking				
Partner	41 (15.8)	68 (26.3)	109 (42.1)	>0.001
Other relatives	14 (5.4)	21 (8.1)	35 (13.5)	>0.001
Work	5 (1.9)	20 (7.7)	25 (9.7)	>0.001
Other social places (restaurants, cafes etc.)	13 (5)	77 (29.7)	90 (34.7)	>0.001

## Discussion

Our study has identified that the initial rate of tobacco use among pregnant women sampled was higher than that of the general population of women in Greece in the same age range (3.7 % of women aged 18–34 smoke in Greece as reported in recent studies) [4, 5]. Addressing smoking in women who are considering pregnancy and targeting women in early pregnancy is a key public health priority for Greece if maternal and infant health outcomes are to be improved. Based on our findings, approximately 73.7 % of women chose not to smoke during pregnancy with 21.7 % reporting quitting in association with their pregnancy. Among woman who continued to smoke during pregnancy, just over half were unable to stop smoking, a quarter did not want to and a small number of women contended they did not believe it was essential, with significant differences in terms of impact of smoking on development of fetal health issues and newborn health problems.

Another criterion that we took into account and on which there was also a statistically significant difference, was the smoking status of the partner [2, 8, 23]. Previous studies which have investigated smoking in pregnancy have also reported that women who did not quit smoking during their pregnancy typically had family members, who were smokers, had partners who smoked, or lived with relatives who smoked [2, 11, 12]. Partners play an important role in influencing women’s smoking behaviour in the perinatal period, and their support (or lack of support) can be an important barrier or facilitator to quitting [2, 8, 23]. A partner who continues using tobacco throughout a woman’s pregnancy is a significant predictor of the current smoking status of the pregnant woman [2, 8, 23]. Moreover, maternal passive smoke exposure during pregnancy has also been shown to have adverse effects on fetal health [8]. Second hand smoke (SHS) exposure during pregnancy is associated with multiple health concerns in the perinatal period including preterm birth, bronchopulmonary dysplasia, wheezing and asthma [8, 23–26].

In our study, despite the high level of awareness that pregnant smokers generally demonstrated about risks to the health of their infants as a consequence of their reluctance to quit smoking, only one third of participants were successful in quitting [2]. Based on our findings, even in cases where women managed to quit smoking or reduce their smoking in pregnancy, they continued to be exposed to passive smoke. Moreover, this occurred either as a consequence of the smoking behavior of their partners and other family members, or as a result of being in social places, such as restaurants [2, 8, 23–26]. In line with previous study findings, our study also found that the two most prominent factors influencing the exposure of women to passive smoking were dining at restaurants (41.6 %) and having a partner who smoked (35.9 %) [2, 8, 23–26]. Having a partner who does not smoke or who quits when the woman becomes pregnant is clearly of benefit to support a pregnant women’s attempts to avoiding contact with passive smoking [2, 8, 23–26].

Although in our study benefits to infant health did not appear to be a motivating factor for other family members to quit smoking, infant health was the most critical reason for pregnant women to quit. Specifically, most of the quitters in our study stopped smoking as soon as their pregnancy was confirmed. It has previously been found that specific psychosocial interventions targeting smoking cessation can increase the number of women who stop smoking in pregnancy, and subsequently reduce low birth weight and preterm births [27]. It is therefore essential that pregnant women, their partners and close relatives are educated on the health risks of active and passive smoking and how these could affect fetal and infant health as well as their own health [2, 8, 23–26, 28]. Moreover, the parents’ social support network, including close family members should be involved in supporting smoke-free environments in spaces shared by the newborn. Strategies for successfully engaging families during the perinatal period should be adopted by community based health professionals including community midwives. Health workers should assist women and their families with addressing SHS exposure during the perinatal period by supporting home smoking bans and reducing infant contact with smokers.

In the current study, most pregnant smokers claimed to have actively tried to quit during their pregnancy but unfortunately just over half did not succeed. A possible reason could be that compared to other quitters, pregnant smokers generally had a longer history of smoking [2, 27]. Furthermore, when advising on quitting, motivational and behavioral support should be provided in parallel with easy access to smoking cessation clinics [27]. It would be also beneficial if this service could be provided in the same maternity hospital or in the community health center.

Whether or not a pregnancy was desired and planned, was also a factor that seemed to affect the willingness of pregnant smokers to quit in our study. Women with planned pregnancies were half as likely to be smokers just before pregnancy, and more likely to give up or reduce the volume of cigarettes as pregnancy progressed. However, unplanned pregnancies had 24 % increased odds of low birth weight and prematurity, compared to planned pregnancies independent of smoking status [17].

Recent studies have reported a number of psychosocial differences between smokers and non-smokers during pregnancy and the postnatal period [29–31]. In our study, women who smoked had significantly higher levels of depressive/anxiety symptoms (Fig. 2) than non-smokers as assessed using the EPDS scale, although caution should be applied to these findings which could reflect women’s experiences of transitory psychological symptoms and/or changes in their functioning and mental state as a normal response to the pregnancy and birth experience. Maternal anxiety and stress may inhibit smoking cessation during pregnancy and promote a relapse after pregnancy in women who have achieved abstinence [30]. Smoking cessation is correlated with depressive symptomatology and should be supported under medical guidance among those smokers who are identified as having mental health symptoms. Community midwives were most likely to provide smoking cessation advice in the study by McCurry et al. 2002 [32]. Moreover, counseling by midwives and healthcare staff were found to significantly reduce the volume of smoking during pregnancy and consequently boost an increase in birth weight [1, 27]. Thus, specific training of community midwives in smoking cessation interventions is needed in order to develop their capability and capacity to provide appropriate and tailored support to pregnant smokers and reduce relapse rates during the postnatal period. In a study from the west of Scotland the development of a home-based midwifery intervention program to support young pregnant smokers to quit was found to be a promising approach to engage young pregnant smokers to help them quit. Local obstetricians and midwives were found to be very willing to support this approach [33].

Smoking during pregnancy not only impacts on the woman’s health, but also on the health of her unborn child. Partners and families of pregnant women should be made aware of this risk and encouraged to participate in smoking cessation programs in order to enhance efforts and quitting results. There is evidence that stopping smoking as early as possible during the pregnancy can reduce the above mentioned risks [34, 35]. Group interventions that include health education information about the risks of smoking and advice to quit, are highly recommended during the perinatal period for smoking cessation support or advice on how to make this change [27]. Women who have had a smoke free pregnancy should be offered help to remain smoke free after birth [34, 35], given that women who quit smoking during pregnancy remain a high-risk group for smoking relapse during the postpartum period [36].

This study had limitations which should be considered. First, maternal smoking status was assessed based on retrospective self-report and without any further clinical assessment. Secondly, we did not follow women up beyond the first 3 days postnatally to assess if pregnant quitters returned to active smoking and women were not routinely asked in early pregnancy about their smoking status in both maternity settings. Moreover, only women who lived in urban areas were able to access the limited free smoking cessation support services offered (mostly in teaching hospitals), if they wanted to quit. As this was a study relevant to perinatal smoking cessation services in Greece, findings may not be not applicable for countries where perinatal smoking cessation services have already been implemented.

Nevertheless, smoking cessation services provided by qualified personnel should be routinely offered in maternity units in Greece. It was apparent in our population that although advice was offered, most pregnant women were unaware or did not know how to access the smoking cessation clinics, which were based in the general hospitals. Although in Greece smoking is banned in public places, there are currently no effective ways of implementing the law. Significant aspects of exposure to passive smoking, such as smoking in cars when children are present, are underestimated and not banned.

The study sample only included participants who gave birth in a public hospital and excluded women who gave birth in private hospitals or at home. However, efforts were made to recruit a representative sample although it should be noted that the rapid socio-economic changes over the last three decades in Greece, have resulted in a relatively homogenous maternal population. Of note is that the majority of women who use public maternity hospitals are routinely transferred to units in Athens for the birth and more specifically to the two large metropolitan university hospitals included in our study. Despite these limitations our sample size is considered satisfactory for statistical analysis.

Other study limitations included the lack of blinding of the midwife researcher to the smoking status of participants which could have potentially resulted in observer bias. Furthermore, there were inherent difficulties due to the low literacy level of women in using a Likert response format. It is possible that other characteristics of maternal smoking behavior corresponded to the differences in risk perceptions and smoking attitudes between smokers and quitters. In this study, we relied on retrospective maternal reports of smoking behavior and recall bias may also have impacted our findings.

The study focused primarily on a potential association between depressive and anxiety symptoms as assessed using the EPDS and maternal active and passive smoking. The EPDS was used in this study because it is a widely administered research tool and no other validated tools are currently available for screening maternal depressive and anxiety symptomology in the Greek language. An EPDS cut-off point of 8/9 was used for screening purposes, in which sensitivity is higher than specificity, in order to detect more potential cases [22].

As only sociodemographic and perinatal variables were assessed as potential confounding factors it is possible that there are other biological and environmental confounding variables which were not detected in this study.

## Conclusion

In this study considerably high rates of tobacco use were reported in the first trimester of pregnancy. Most women chose to stop smoking although there were high levels of exposure to passive smoking. Perceptions of fetal health risks and attitudes towards smoking during pregnancy were critical prognostic factors of the anti-smoking behaviors of pregnant women in the study sample. Our data supports the importance of ensuring that pregnant women, their partners and close relatives are educated on the health risks of active and passive smoking and how these could have an adverse effect on birth and other health outcomes of women infants. Smoke-free environments are necessary to promote perinatal maternal and infant health. There is an emerging need to highlight the international aspects of this critical public health issue.
